# Receptor for advanced glycation end-products and ARDS prediction: a multicentre observational study

**DOI:** 10.1038/s41598-018-20994-x

**Published:** 2018-02-08

**Authors:** Matthieu Jabaudon, Pauline Berthelin, Thibaut Pranal, Laurence Roszyk, Thomas Godet, Jean-Sébastien Faure, Russell Chabanne, Nathanael Eisenmann, Alexandre Lautrette, Corinne Belville, Raiko Blondonnet, Sophie Cayot, Thierry Gillart, Julien Pascal, Yvan Skrzypczak, Bertrand Souweine, Loic Blanchon, Vincent Sapin, Bruno Pereira, Jean-Michel Constantin

**Affiliations:** 1 CHU Clermont-Ferrand, Department of Perioperative Medicine, Clermont-Ferrand, France; 2 Université Clermont Auvergne, CNRS UMR 6293, INSERM U1103 GReD Clermont-Ferrand, France; 3 CHU Clermont-Ferrand, Department of Medical Biochemistry and Molecular Biology, Clermont-Ferrand, France; 4 Jean Perrin Comprehensive Cancer Center, Department of Anesthesiology and Critical Care Medicine, Clermont-Ferrand, France; 5 CHU Clermont-Ferrand, Medical Intensive Care Unit, Université Clermont Auvergne, UMR CNRS 6023 Clermont-Ferrand, France; 6 CHU Clermont-Ferrand, Biostatistical Unit, Department of Clinical Research and Innovation (DRCI), Clermont-Ferrand, France

## Abstract

Acute respiratory distress syndrome (ARDS) prediction remains challenging despite available clinical scores. To assess soluble receptor for advanced glycation end-products (sRAGE), a marker of lung epithelial injury, as a predictor of ARDS in a high-risk population, adult patients with at least one ARDS risk factor upon admission to participating intensive care units (ICUs) were enrolled in a multicentre, prospective study between June 2014 and January 2015. Plasma sRAGE and endogenous secretory RAGE (esRAGE) were measured at baseline (ICU admission) and 24 hours later (day one). Four *AGER* candidate single nucleotide polymorphisms (SNPs) were also assayed because of previous reports of functionality (rs1800625, rs1800624, rs3134940, and rs2070600). The primary outcome was ARDS development within seven days. Of 500 patients enrolled, 464 patients were analysed, and 59 developed ARDS by day seven. Higher baseline and day one plasma sRAGE, but not esRAGE, were independently associated with increased ARDS risk. *AGER* SNP rs2070600 (Ser/Ser) was associated with increased ARDS risk and higher plasma sRAGE in this cohort, although confirmatory studies are needed to assess the role of *AGER* SNPs in ARDS prediction. These findings suggest that among at-risk ICU patients, higher plasma sRAGE may identify those who are more likely to develop ARDS.

## Introduction

Acute respiratory distress syndrome (ARDS) still carries a high mortality rate^1,2^, and a major challenge in targeting the prevention and early treatment of ARDS is the inability to accurately predict which patients will develop the syndrome^3–6^. The Lung Injury Prediction Score (LIPS) was developed to identify patients at high risk of developing ARDS; however, its positive predictive value is limited (0.14–0.23)^7^. To improve prediction, one method may be to combine clinical data with plasma biomarkers that reflect the pathogenesis of ARDS such as angiopoietin-2 (Ang-2), a marker of lung endothelial injury^8^. In contrast, the soluble receptor for advanced glycation end-products (sRAGE), a marker of lung epithelial injury^9^, may predict ARDS more accurately in selected at-risk patients, e.g. after cardiac surgery^10^, severe trauma^11^, or after major surgery^12^. Soluble forms of RAGE (sRAGE) include the extracellular domain of membrane RAGE that is cleaved by proteinases^13^ and endogenous secretory RAGE (esRAGE), among other forms produced by alternative splicing^14,15^. sRAGE has good diagnostic value for ARDS and is associated with lung injury severity, the degree of lung epithelial injury, impaired alveolar fluid clearance, and prognosis in ARDS^16,17^. Although it remains unclear whether esRAGE may be involved in ligand binding or may have any functional effects by itself, esRAGE levels were lower in the plasma and alveolar fluid from patients with ARDS than in mechanically ventilated controls without ARDS in a previous study^18^. In addition, and although not yet ready for clinical use, genomic applications could facilitate better prediction, diagnosis, disease subclassification, and prognosis for ARDS^19–22^, but the association of single nucleotide polymorphisms (SNPs) flanking the *AGER* gene with susceptibility to ARDS remains unknown.

Because epithelial injury is a major contributor of ARDS pathogenesis, resolution, and prognosis^23,24^, we hypothesised that plasma levels of sRAGE and *AGER* SNPs might predict the development of ARDS in a high-risk population of patients admitted to intensive care units (ICUs).

Some of the results of this study have been previously reported in the form of an abstract during the *American Thoracic Society International Conference (2017)*.

## Results

### Baseline characteristics

**Figure 1:**
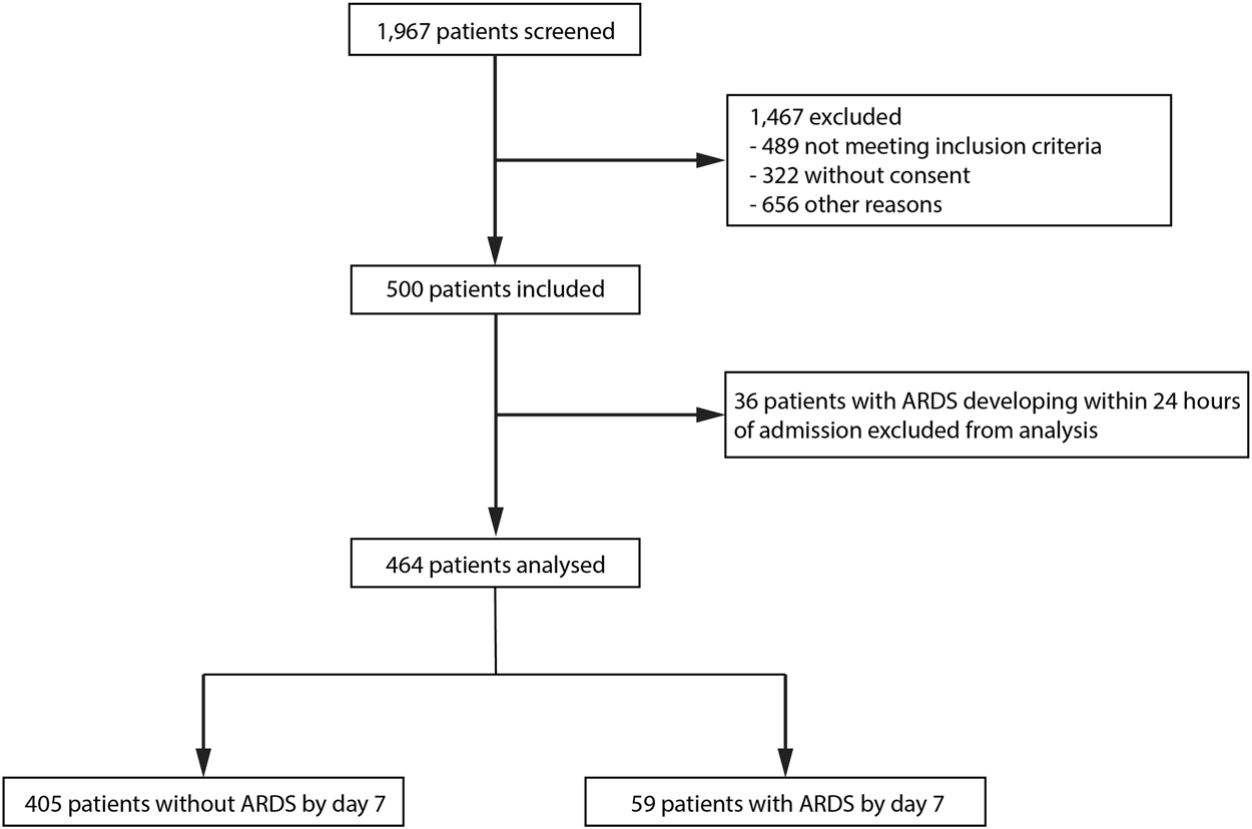
Flow diagram of the study. Personnel shortage was another identified reason for non-enrollment of eligible subjects, and some subjects were missed without a given explanation.

Between June 2014 and January 2015, 1,967 patients were screened. A total of 500 patients were enrolled and analysed, among whom 464 patients did not meet the criteria for ARDS within 24 hours (Fig. 1, Table 1). Patients who developed ARDS by day seven (n = 59, 13%) were more severely ill, with higher simplified acute physiology scores II (SAPS II) at ICU admission (baseline) than patients who did not develop ARDS (n = 405). Plasma samples were drawn a median of 4.2 ± 1.8 hours after ICU admission. 18 patients (4%) developed ARDS between days seven and 30, and had similar plasma sRAGE and esRAGE than those who did not.

**Table 1:** The baseline characteristics of patients who developed acute respiratory distress syndrome (ARDS) (n = 59) or did not develop ARDS (n = 405) by day seven.

	No ARDS (n = 405)	Develop ARDS (n = 59)	p-value
Age (years)	61 ± 16	62 ± 16	0.5
Male sex	267 (66)	46 (78)	0.07
Race/ethnicity			0.2
- White	389 (96)	54 (92)	
- Black	12 (3)	4 (7)	
- Asian	4 (1)	1 (1)	
Body mass index (kg/m^2^)	26.6 ± 6.2	26.9 ± 6.5	0.9
Primary admission diagnosis			
- Cardiac	8 (2)	2 (3)	0.6
- Respiratory	251 (62)	38 (65)	0.6
- Gastrointestinal	69 (17)	11 (17)	0.9
- Infectious	117 (29)	22 (37)	0.2
- Neurological	56 (14)	9 (15)	0.8
- Major surgery	101 (25)	14 (23)	0.7
- Other	20 (5)	2 (3)	0.7
Coexisting chronic conditions			
- Atherosclerosis	89 (22)	15 (25)	0.6
- Diabetes	73 (18)	8 (14)	0.5
- Hypertension	162 (40)	27 (46)	0.5
- Dyslipidemia	85 (21)	15 (25)	0.5
- Current smoking	101 (25)	19 (32)	0.3
- Asthma	12 (3)	2 (3)	0.9
- COPD	41 (10)	10 (17)	0.2
- Chronic renal failure requiring dialysis	12 (3)	1 (2)	0.7
- Liver cirrhosis	20 (5)	2 (3)	0.8
- Cancer	77 (19)	14 (22)	0.6
ARDS risk factor			
- Shock	101 (25)	16 (27)	0.8
- Sepsis	113 (28)	20 (34)	0.3
- Pneumonia	126 (31)	29 (49)	0.1
- Aspiration	24 (6)	5 (8)	0.9
- Severe trauma	41 (10)	5 (8)	0.9
- Pancreatitis	24 (6)	23 (5)	0.9
- Drug overdose	32 (8)	4 (6)	0.7
- High-risk surgery	89 (22)	9 (14)	0.8
Lung Injury Prediction Score (LIPS)	4.9 ± 2.4	5.7 ± 2.8	0.07
Simplified Acute Physiology Score II	43 ± 19	49 ± 18	0.01
Vasopressor use at admission	93 (23)	17 (29)	0.5
Invasive ventilation at admission	186 (46)	34 (58)	0.09
Noninvasive ventilation at admission	28 (7)	3 (5)	0.8
30-day mortality	49 (12)	11 (19)	0.003

The data are presented as mean ± standard deviation or n (%). Analyses were performed using the Wilcoxon rank-sum, a chi-square test, or Fisher’s exact test, as appropriate. Percentages may not exactly total 100% because of rounding. *COPD: chronic obstructive pulmonary disease*.

### The predictive value of the soluble forms of RAGE for developing ARDS

**Figure 2:**
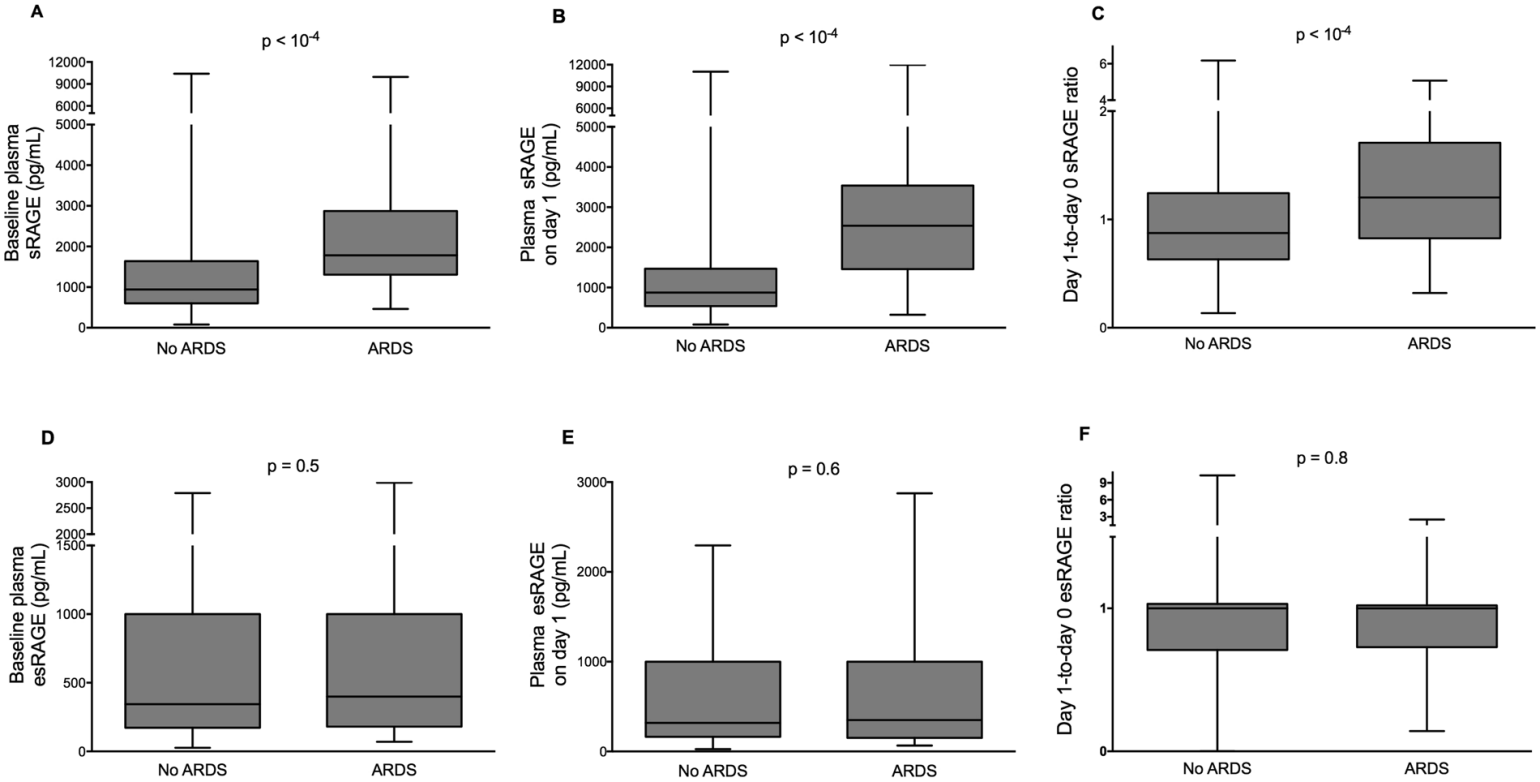
Plasma biomarker levels according to ARDS development. Patients who developed ARDS at least 24 hours after the first sample draw (n = 59) had statistically significant increased (**A)** baseline plasma sRAGE, (**B)** plasma sRAGE on day one, and (**C)** day one-to-day zero plasma sRAGE ratio than those who did not develop ARDS (n = 405). (**D**) Baseline plasma esRAGE, (**E**) plasma esRAGE on day one, and (**F)** day one-to-day zero plasma esRAGE ratio were similar between groups.

**Figure 3:**
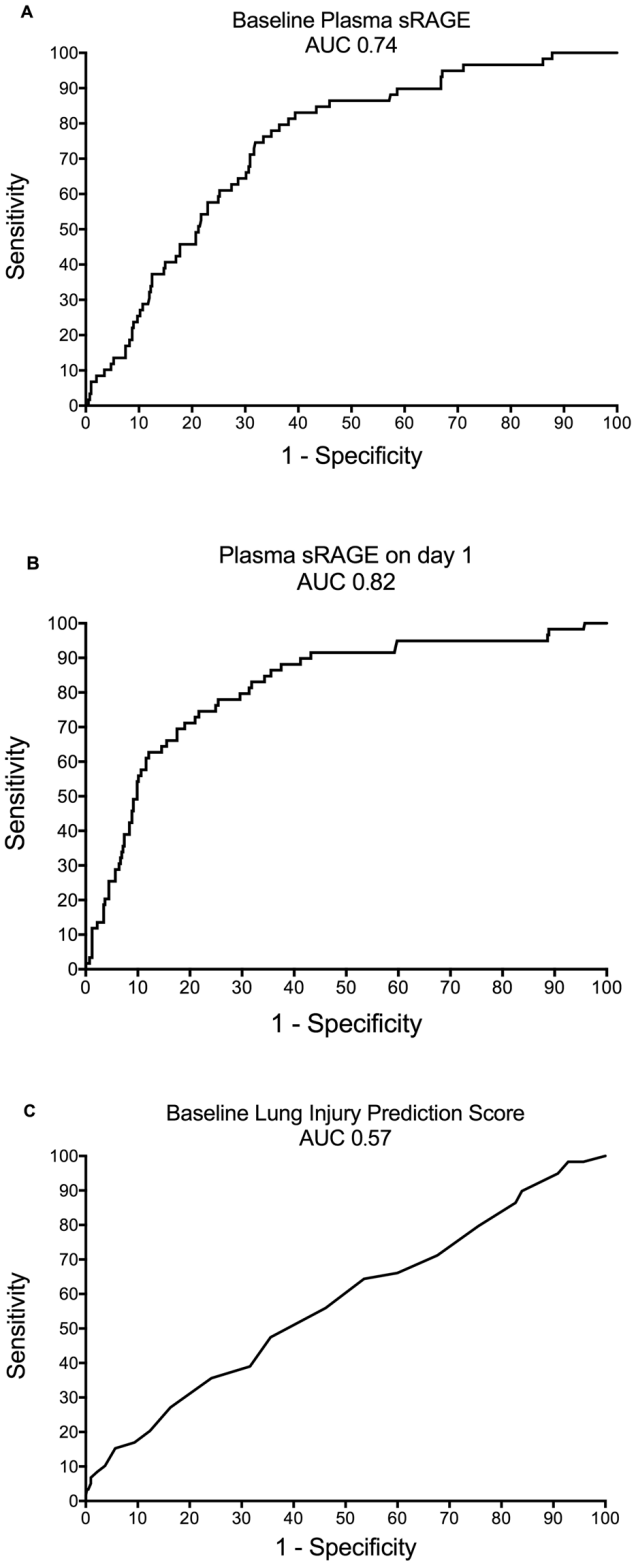
Receiver operating characteristic (ROC) curves. (**A)** Baseline plasma sRAGE (area under ROC curve (AUROC), 0.74; 95% CI, [0.68–0.80] and (**B)** plasma sRAGE on day one (AUROC, 0.82 [0.76–0.88]) each showed good discrimination between those who developed ARDS and those who did not, but (**C)** the LIPS was poorly discriminative in this population of patients at risk of developing ARDS (AUROC, 0.57 [0.49–0.65]).

Plasma sRAGE was significantly higher, at baseline and on day one, among patients who developed ARDS by day seven than among those who did not (Fig. 2A–C). In contrast, esRAGE at baseline and on day one was similar in both groups (Fig. 2D–F). There was significant correlation between plasma sRAGE levels measured at baseline and those measured on day one (Spearman’s rho = 0.72, 95% confidence interval 0.67–0.75, p < 10^−3^) (see Supplementary Fig. S1). To evaluate the discrimination of the models based on sRAGE and esRAGE, we calculated the area under a receiver operating characteristic curve (AUROC). Plasma sRAGE at baseline and on day one had good discrimination, with AUROC of 0.74 (95% confidence interval (CI), [0.68–0.80]) (Fig. 3A) and 0.82 [0.76–0.88] (Fig. 3B), respectively. The day one-to-day zero plasma sRAGE ratio, the baseline plasma sRAGE-to-esRAGE ratio, and the day one plasma sRAGE-to-esRAGE ratio had AUROCs of 0.66 [0.58–0.73], 0.62 [0.54–0.71], and 0.69 [0.61–0.78], respectively. The LIPS showed poor discrimination (AUROC, 0.57 [0.49–0.65]; Fig. 3C). Using conventional methods, thresholds for sRAGE were set at 1,340 pg/mL at baseline and 1,096 pg/mL on day one. At these thresholds, the plasma sRAGE at baseline and on day one had sensitivities, specificities, positive (PPV) and negative predictive values (NPV) of 75%, 68%, 24%, and 95% and 86%, 64%, 25%, and 97%, respectively, for ARDS development.

### The predictive value of *AGER* gene polymorphisms for developing ARDS

Allele frequencies were in Hardy–Weinberg equilibrium (HWE) for all candidate SNPs in the whole cohort and in both subpopulations of patients with and without ARDS by day seven (Supplement, Table S1).

The *AGER* SNPs rs1800625, rs1800624 and rs3134940 (either homozygous or heterozygous) were distributed similarly between patients who developed ARDS by day seven and those who did not (p = 0.6 for all). In contrast, ARDS was more frequent with than without SNP rs2070600 (32% vs. 12%, respectively, p < 10^−3^). In particular, homozygous rs2070600 (the Ser/Ser genotype) was associated with a higher risk of developing ARDS (92%) than heterozygous rs2070600 (the Gly/Ser genotype) (7%) or without the SNP (13%, p < 10^−3^). Therefore, only the Ser/Ser genotype was included in subsequent multivariate analyses. Patients with the Ser/Ser genotype had higher baseline plasma sRAGE than those without the SNP (median [IQR], 1370 [846–2,059] vs. 1013 [627–1,788] pg/mL, p = 0.035). The frequency of rs1800625, rs1800624, rs3134940, and rs2070600 did not differ between patients who developed ARDS after day seven and those who did not (p = 0.9, 0.5, 0.8, and 0.4, respectively).

### Multivariate adjustments of the predictor models

Baseline plasma sRAGE, the plasma sRAGE on day one, and the day one-to-day zero plasma sRAGE ratio predicted ARDS development (OR, 2.25 [1.60–3.16], 4.33 [2.85–6.56], and 1.61 [1.17–2.22], respectively), even after adjustment for SAPS II and the presence of sepsis, shock, or pneumonia at baseline (Table 2). Both baseline plasma sRAGE and the day one-to-day zero plasma sRAGE ratio were independent predictors of ARDS development (OR, 3.21 [2.17–2.22] and 2.52 [1.73–3.67], respectively) (Table 2).

**Table 2:** The associations between soluble RAGE levels and the prediction of ARDS by day seven in multivariate analyses.

Models	OR	95% CI	p-value
**Baseline sRAGE**	2.25	[1.60–3.16]	<10^−3^
SAPS II	1.02	[1.00–1.03]	0.04
Sepsis	1.34	[0.65–2.78]	0.4
Shock	0.87	[0.43–1.74]	0.7
Pneumonia	1.55	[0.67–3.58]	0.3
**Plasma sRAGE on day one**	4.33	[2.85–6.56]	<10^−3^
SAPS II	1.01	[0.99–1.03]	0.2
Sepsis	1.21	[0.56–2.63]	0.6
Shock	0.8	[0.39–1.65]	0.6
Pneumonia	1.35	[0.56–3.26]	0.5
**Day one-to-day zero sRAGE ratio**	1.61	[1.17–2.22]	0.004
SAPS II	1.02	[0.99–1.03]	0.07
Sepsis	1.28	[0.63–2.61]	0.5
Shock	0.82	[0.42–1.63]	0.6
Pneumonia	2.1	[0.93–4.74]	0.07
**Baseline sRAGE**	3.21	[2.17–4.75]	<10^−3^
Day one-to-day zero sRAGE ratio	2.52	[1.73–3.67]	<10^−3^
SAPS II	1.01	[0.99–1.03]	0.2
Sepsis	1.32	[0.62–2.81]	0.5
Shock	0.82	[0.40–1.69]	0.6
Pneumonia	1.58	[0.67–3.73]	0.3

The analyses were adjusted for baseline severity (as assessed by SAPS II) and the presence of sepsis, shock, or pneumonia at baseline. Plasma sRAGE levels (in pg/mL) are natural log-transformed in the logistic regression model to meet the assumption of linearity with log-odds of outcome; the ORs presented here are for each log increase in the level of plasma sRAGE.

Both higher baseline plasma sRAGE and SNP rs2070600 were associated with an increased risk of developing ARDS (OR, 2.39 [1.65–3.45] and 124.59 [14.89–1,043.34], respectively) after multivariate adjustment, even when used together for multivariate analyses (Supplement, Table S2). Using this model, 90% of patients were correctly classified as being at risk of developing ARDS or not (AUROC, 0.81 [0.75–0.88]; sensitivity, 20%; specificity, 100%; PPV, 92%; NPV, 90%).

### Analysis of time to ARDS as a censored variable

**Figure 4:**
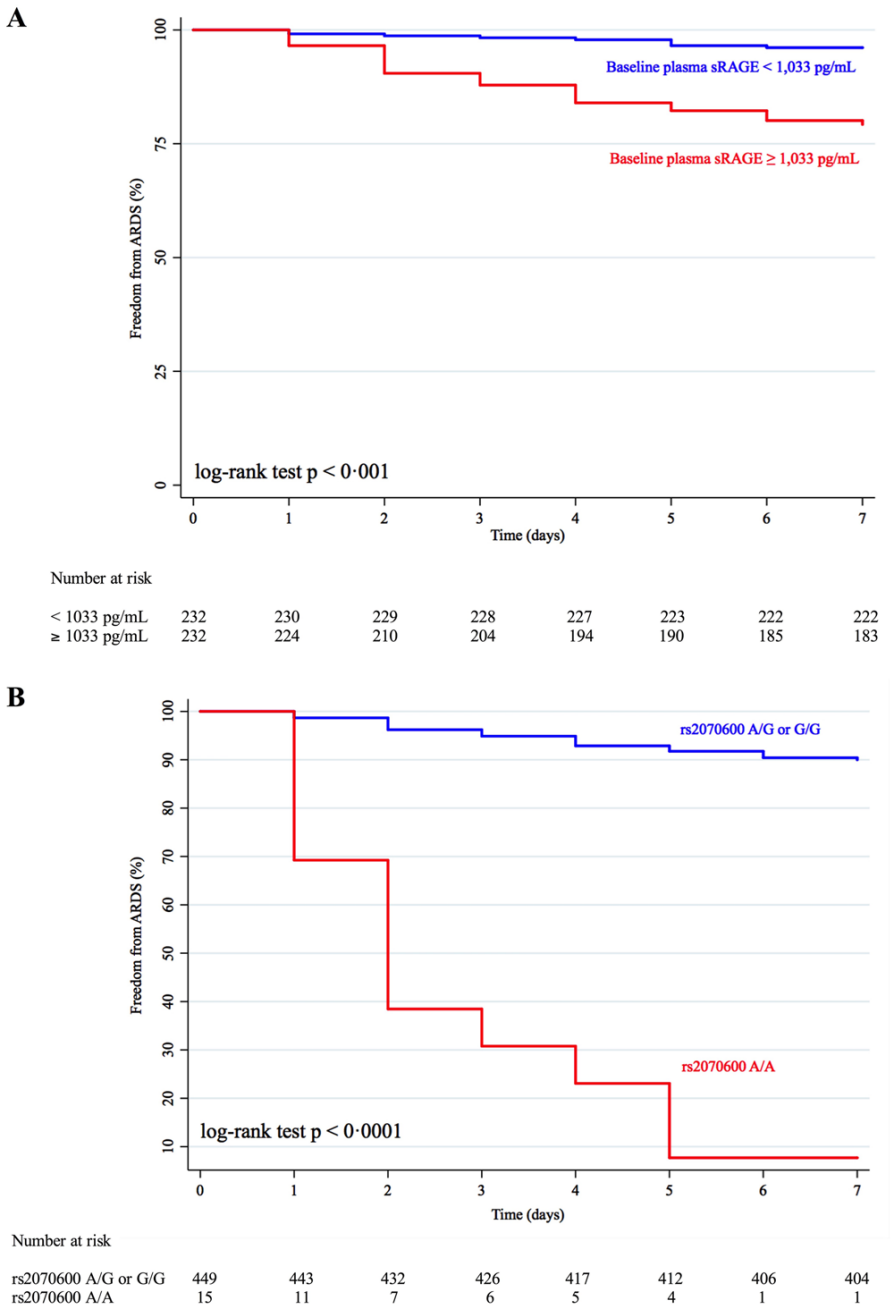
The cumulative proportion of patients who did not develop ARDS within seven days of admission to the ICU for (**A)** patients with baseline plasma sRAGE above or below 1,033 pg/mL (the median value of baseline plasma sRAGE in patients from our cohort) and (**B)** patients with or without homozygous SNP rs2070600 (the Ser/Ser genotype) within the gene coding for RAGE.

Using the same covariates as in previous multivariate models, the HR for developing ARDS within seven days, when baseline plasma sRAGE was above 1,033 pg/mL (its median value in our cohort), was 4.59 [1.47–14.37] (Fig. 4A). The rs2070600 SNP was associated with a HR for developing ARDS of 15.09 [6.33–35.99] (Fig. 4B) (Supplement, Table S3).

### Sensitivity analyses

Analyses were repeated only in patients under invasive mechanical ventilation at baseline (n = 222), of whom 34 (15%) developed ARDS within seven days. Plasma sRAGE was higher in patients who developed ARDS than in those who did not (p < 10^−4^ at baseline and on day one). Baseline plasma sRAGE (AUROC, 0.71 [0.63–0.79]) and sRAGE on day one (AUROC, 0.79 [0.70–0.87]) had good discrimination in this subset, whereas the LIPS showed poor discrimination (AUROC, 0.57 [0.46–0.69]). The AUROC for the day one-to-day zero plasma sRAGE ratio was 0.63 [0.53–0.73].

After adjusting for respiratory parameters (tidal volume, level of positive end-expiratory pressure, and inspiratory plateau pressure), SAPS II, and PaO_2_/FiO_2_ and the presence of sepsis, shock, or pneumonia at baseline, higher baseline sRAGE, and SNP rs2070600 remained associated with an increased risk of developing ARDS (Supplement, Table S4).

## Discussion

In this sample of critically ill at-risk patients, plasma sRAGE, when measured upon ICU admission or on day one, could predict the development of ARDS within seven days. In contrast, plasma esRAGE did not predict ARDS. In addition, and although these results should be interpreted carefully, *AGER* SNP rs2070600 (the Ser/Ser genotype) was associated with a higher risk of developing ARDS and with higher plasma sRAGE.

Because ARDS mortality remains high^2^, current initiatives should include primary prevention^4,6,25^. However, a key challenge is to identify at-risk patients in whom ARDS is likely to develop and who would benefit if ARDS were prevented. Although clinical scores can identify patients who are more likely to develop ARDS^7^, biomarkers may improve the predictive value of the clinical-only LIPS in pre-identified at-risk populations^8^. In our high-risk cohort (mean LIPS, 5 ± 2.5), the plasma sRAGE was particularly adept at ARDS prediction, with better test characteristics than LIPS or a biomarker (Ang-2) when used in a population of unselected ICU patients^8^. Because personalized approaches can decrease the incidence of ARDS^26^, the identification of predictive clinical or biological variables is of major importance to developing preventive strategies, early detection, and treatment^4,6^. Our findings may be important not only because they provide an approach to ARDS prediction but also because they provide new insights into the implications of RAGE pathway in the early pathogenesis of ARDS, with increased plasma levels of sRAGE, a marker of lung epithelial injury and of impaired alveolar fluid clearance^9,17,27,28^, and potential genetic susceptibility driven by rs2070600. Because epithelial injury is a major mechanism of lung injury, this hypothesis is further supported by the good predictive value of plasma sRAGE on day one, suggesting that the kinetics of sRAGE itself may better identify patients at greatest risk. However, baseline and day one measurements of sRAGE were significantly correlated in our cohort and may provide similar overall prediction. Moreover, evidence is mounting to support the role of both sRAGE and esRAGE as biomarkers of ligand–RAGE activity in diseases or endogenous protection factors against RAGE-mediated pathogenesis^29^. In our cohort, the predictive value of plasma esRAGE was poor, although the sRAGE-to-esRAGE ratio remained informative, in line with previous findings in other lung diseases^30^. sRAGE comprises a heterogeneous population including esRAGE, a soluble splice variant, and its proteolytically cleaved forms shed into the bloodstream by metalloproteinases^13^. In general, plasma esRAGE is between two- and five-fold lower than plasma sRAGE, and plasma esRAGE only explains about one-third of the variation in plasma sRAGE, suggesting specific kinetics^31^. In addition, the balance in the respective levels of membrane-bound RAGE, esRAGE, and sRAGE, may be of primary importance in controlling RAGE-dependent actions, thus reinforcing the value of sRAGE as a biomarker in ARDS^16–18,32^.

Although exploratory and more hypothesis-generating than definitive, our findings support a novel association between rs2070600 (the Ser/Ser genotype) and an increased risk of developing ARDS. The somewhat more common (G) allele encodes for glycine (Gly), while the minor (A) allele encodes for serine (Ser). Although the exchange of glycine for serine amino acids within RAGE protein is unlikely to impact its secondary or three-dimensional structure (see Supplementary Fig. S2), it may modify cell surface expression of RAGE, ligand-affinity, and pro-inflammatory signalling^33,34^. Future studies are needed to confirm these results and to better understand the precise mechanisms through which rs2070600 predisposes at-risk patients for developing ARDS. Previous studies have found an association of *AGER* gene variants with enhanced susceptibility to RAGE-mediated pathogenesis^35^, and preliminary results suggest that plasma sRAGE may be, at least in part, under genetic control. In our study, rs2070600 was associated with higher plasma sRAGE, thus contrasting with the previous reports of lower sRAGE in healthy nondiabetic, nonobese, non-critically ill patients^36^. Our findings also prompt further research on differential relationships between plasma sRAGE and *AGER* SNPs in ARDS vs. other respiratory diseases such as chronic obstructive pulmonary disease (COPD)^37^, in which RAGE expression seems mostly downregulated. Understanding the respective contributions of the genetic control of *AGER* expression, of its positive feedback following ligand accumulation, and of proteolytic cleavage of membrane RAGE by metalloproteinases^13^, among other factors, to circulating sRAGE levels in both acute and chronic conditions would necessitate specific measurements (e.g., plasma MMP9, ADAM10) that were out of the scope of the current study and deserve further studies.

Our study had some limitations. First, our findings may only hold true in patients with a high-risk of developing ARDS, as assessed by elevated LIPS, but not in all critically ill patients^8^. Therefore, the identification of clinical risk factors remains a cornerstone in the prediction of ARDS^7^. Second, because a derivation cohort may demonstrate better biomarker test characteristics than a validation cohort, replicating our results in an independent cohort of patients remains necessary. Third, we cannot say whether elevated plasma sRAGE may be a better ARDS predictor in patients with pulmonary risk factors (e.g. pneumonia and aspiration, which were predominant in our cohort) than in those with non-pulmonary risk factors. However, direct injury to the lung is the primary cause of ARDS in most patients and our results may further support an endotype consistent with more severe lung epithelial injury in direct ARDS^38^. Fourth, although sRAGE, a marker of lung epithelial injury, may be informative to discriminate patients at higher risk of developing ARDS, the predictive value of other markers, such as Ang-2 (marker of lung endothelial injury) or markers of a hyperinflammatory phenotype (interleukin (IL)−8, tumor necrosis factor-receptor 1, bicarbonates^39^, and/or IL-6, interferon gamma, angiopoietin 1/2 and plasminogen activator inhibitor-1^40^), alone or combined together, deserves further investigation. Fifth, there is no point-of-care test for sRAGE at this time, and genomic applications are not yet ready for clinical use, which currently limits the potential application of our findings in clinical medicine^22^ or to enrich future clinical trials^41^. If sRAGE is confirmed as a useful ARDS predictor, further research will be needed to make rapid assays more immediately available to clinicians. Last, our findings on the association between *AGER* SNP rs2070600 and the risk of developing ARDS should be interpreted with caution. Our genotyping strategy was limited to four candidate *AGER* SNPs only, and other variation in *AGER* remains unexplored. In addition, statistical power was primarily calculated for differences in baseline plasma sRAGE, not for these exploratory genetic association studies. Of note, our study also lacked a validation cohort in which to verify the use of sRAGE and *AGER* gene variants for the prediction of ARDS. However, because both subpopulations of patients with and without ARDS by day seven are in HWE for rs2070600, then it is more likely that difference in allele frequencies of the two subpopulations is due to reproductive isolation rather than selection bias.

Our study also had several unique strengths. First, it was designed specifically for the early collection of blood samples, allowing us to capture many at-risk patients before they developed ARDS. This is a difficult population to recruit, because most patients present with ARDS, but it is also the population that is most likely to benefit from such predictions^25,42^. Second, we rigorously excluded patients with ARDS at admission and those who developed ARDS within the first 24 hours of the study to reduce the likelihood of enrolling patients with established early disease^8^. Third, our study included both intubated and nonintubated patients, with few exclusion criteria, which allowed us to analyze a broad range of critically ill patients with ARDS risk factors; these patients represent a clinically relevant population and their breadth increases the generalisability of our study.

In conclusion, our study is the first to report the predictive value of plasma sRAGE, but not plasma esRAGE, for developing ARDS in a high-risk population of ICU patients. *AGER* SNP rs2070600 (Ser/Ser) was associated with increased ARDS risk and higher plasma sRAGE in this cohort. Although further confirmatory studies are warranted, such findings might open new avenues for the research stratification of at-risk patients in future clinical trials of early therapeutic or preventive approaches to ARDS.

## Methods

### Study design and participants

PrediRAGE (PREDIctive values of plasma soluble RAGE levels and gene polymorphisms for the onset of ARDS in critically ill patients) is an investigator-initiated, multicentre, observational, cohort study undertaken at five ICUs from two hospitals in Clermont-Ferrand, France. The study protocol was approved by the institutional review board *Comité de Protection des Personnes Sud-Est-VI* (AU10732) and the *Comité consultatif sur le traitement de l’information en matière de recherche* (14.017). This study was performed in accordance with the Strengthening the Reporting of Observational studies in Epidemiology (STROBE) statement^43^. The datasets generated during the current study are available from the corresponding author on reasonable request.

Patients aged 18 years or older were eligible if they were admitted to participating ICUs with at least one identified ARDS risk factor (Supplement, Table S5)^1^. All participants, or their next of kin, provided written informed consent. There was no deviation from the approved protocol. This trial was registered with ClinicalTrials.gov, number NCT02070536.

### Biological sample collection and measurements

Plasma specimens were obtained within six hours of ICU admission (day zero) and 24 hours later (day one). Plasma sRAGE and esRAGE were measured blindly in duplicate using commercially-available enzyme-linked immunosorbent assay kits (Human RAGE Quantikine ELISA kit, R&D Systems, MN, USA (detection limit, 78 pg/mL) and Human esRAGE ELISA kit, B-Bridge International, Japan (detection limit, 50 pg/mL), respectively).

According to the results from previous studies of inflammatory conditions^33,35,44,45^, four candidate *AGER* SNPs were chosen *a priori* for genotyping: rs1800625 (the C allele of the _429 T/C polymorphism within the promoter region of the *AGER* gene), rs1800624 (the A allele of the _374 T/A polymorphism within the promoter region), rs3134940 (the A allele of the 2184 A/G polymorphism within intron 8) and rs2070600 (the A allele of the Gly82Ser polymorphism within exon 3). At baseline, a peripheral blood sample was obtained from each participant and DNA was isolated using FlexiGene DNA kit (Qiagen, Venlo, Netherlands). The concentrations of extracted DNAs were measured by NanoDrop ND-1000 spectrophotometer (Thermo Fisher Scientific, Wilmington, DE, USA); any sample with a DNA concentration < 50 ng/µl was excluded and required another sample. DNA genotyping of four selected *AGER* SNPs was performed using the chain termination method (Sanger sequencing) by GATC Biotech (Constance, Germany). Primers for DNA sequencing and polymerase chain reaction are listed in Table S6 (Supplement).

### Primary outcome and additional variables

The primary outcome was the difference in baseline plasma sRAGE between patients who developed ARDS within seven days after enrolment into the study and those who did not.

The primary outcome follow-up time was chosen *a priori* because ARDS criteria must be met by definition within 1 week of a known clinical insult or new or worsening respiratory symptoms^1^. ARDS was defined by physicians (blinded to RAGE levels and *AGER* genotype) caring for the patients, based on criteria from the Berlin definition^1^. Chest radiographs and arterial blood gases were routinely performed in participating ICUs: at least daily, and more frequently if prompted by clinical symptoms (e.g., new or worsening respiratory symptoms).

Patients who met the criteria for ARDS at baseline, before the sample draw, or within the subsequent 24 hours, were excluded to ensure the removal of ARDS that was present at baseline (Fig. 1)^8^. Patients were followed for 30 days for mortality and ARDS developing after day seven.

Secondary endpoints included differences in plasma sRAGE on day one, in plasma esRAGE at baseline and on day one, and in *AGER* SNPs distributions between patients who developed ARDS within seven days and those who did not, and between patients who developed ARDS within 30 days and those who did not.

### Statistical analysis

Based on previous data^8,18^, we calculated *a priori* that a sample of 458 patients (with a minimal ARDS incidence of 8%) would be needed to detect a difference in baseline plasma sRAGE (mean ± standard deviation (SD), 500 ± 1,000 pg/mL) between the patients with ARDS by day seven and those without, with a statistical power of 80%. Thus, we planned to include 500 patients. Qualitative data were expressed as numbers and percentages, and quantitative data as means, SD, or medians and interquartile ranges [IQR]. A Student’s t-test or a Mann-Whitney test were used for quantitative parameters according to t-test assumptions. Categorical data were compared using a chi-square test or Fisher’s exact test. Univariate correlations between quantitative outcomes were assessed using Pearson or Spearman correlation coefficients where appropriate. Then, multivariate logistic generalised linear mixed models (logistic for binary endpoint) were adjusted for potential clinically relevant confounders, which were chosen *a priori* based on a previous study of ARDS prediction by Agrawal *et al*.^8^: sepsis, shock, pneumonia, and SAPS II^46^. Both the plasma levels of sRAGE/esRAGE and gene variants were covariate candidates for multivariate prediction. The centre (ICU) effect was considered a random-effect to account for between- and within-centre variability. Results were expressed as odds ratios (ORs, with a 95% CI) for developing ARDS. Then, time to ARDS onset was considered a censored variable upon the last follow-up at day seven, assuming that patients who had been discharged from the ICU before day seven did not develop ARDS at this time-point. The log-rank test was performed for univariate comparisons, and the Cox proportional-hazards regression was used for multivariate analysis. Results were expressed as hazard ratios (HRs, with a 95% CI) for developing ARDS: 1) in patients with plasma sRAGE above or below its median values in the cohort, and 2) in patients with or without SNPs which were eventually found significant in univariate analysis. Finally, the discrimination was tested by calculating the AUROC and a 95% CI; thresholds were determined according to clinical relevance and usual indexes (e.g. Liu, Youden, efficiency).

Data quality was checked for genetic analyses; allele frequencies, genotype frequencies, and disequilibrium coefficients for codominant traits or data of completely known genotypes were estimated, and asymptotic HWE tests were performed^47^ using Stata command *genhw*^48^. Plasma sRAGE and esRAGE were compared across genotypes using linear regression analyses adjusted for the factors previously defined. When appropriate, normality of dependent variables (e.g., plasma sRAGE) was achieved using logarithmic transformation. Genotype groups were analysed separately and combined together (the homozygous minor allele genotype plus the heterozygous genotype). Sensitivity analyses were planned *a priori* in patients under invasive mechanical ventilation at baseline. In addition to covariates used in previous multivariate models, the use of some clinically relevant respiratory variables (tidal volume, level of positive end-expiratory pressure, and inspiratory plateau pressure, and PaO_2_/FiO_2_) for adjustment of sensitivity analyses was planned *a priori*. A two-sided p-value < 0.05 was considered significant. Statistical analysis was performed with Stata software (v14, StataCorp, College Station, TX, USA).

## Electronic supplementary material

Supplementary Information
