# Supplementary material for: Receptor for advanced glycation end-products and ARDS prediction: a multicentre observational study
Source: Sci Rep. 2018 Feb 8;8:2603. doi: 10.1038/s41598-018-20994-x (PMC5805783; doi:10.1038/s41598-018-20994-x)
Supplement: Supplementary file 1 — Supplementary Information [file 41598_2018_20994_MOESM1_ESM.pdf]

SUPPLEMENTARY INFORMATION

Receptor for advanced glycation end-products and ARDS prediction: a multicentre observational study

Matthieu Jabaudon, MD, PhD, Pauline Berthelin, MD, Thibaut Pranal, MD, Laurence Roszyk, PharmD, Thomas Godet, MD, PhD, Jean-Sébastien Faure, MD, Russell Chabanne, MD, MSc, Nathanael Eisenmann, MD, Alexandre Lautrette, MD, PhD, Corinne Belville, PhD, Raiko Blondonnet, MD, MSc, Sophie Cayot, MD, Thierry Gillart, MD, Julien Pascal, MD, Yvan Skrzypczak, MD, Bertrand Souweine, MD, PhD, Loic Blanchon, PhD, Vincent Sapin, PharmD, PhD, Bruno Pereira, PhD, Jean-Michel Constantin, MD, PhD

SUPPLEMENTARY FIGURES

Supplementary Fig. S1. The correlation between plasma levels of sRAGE as measured at baseline (ICU admission) and 24 hours later (day one). CI: confidence interval. ICU: intensive care unit.

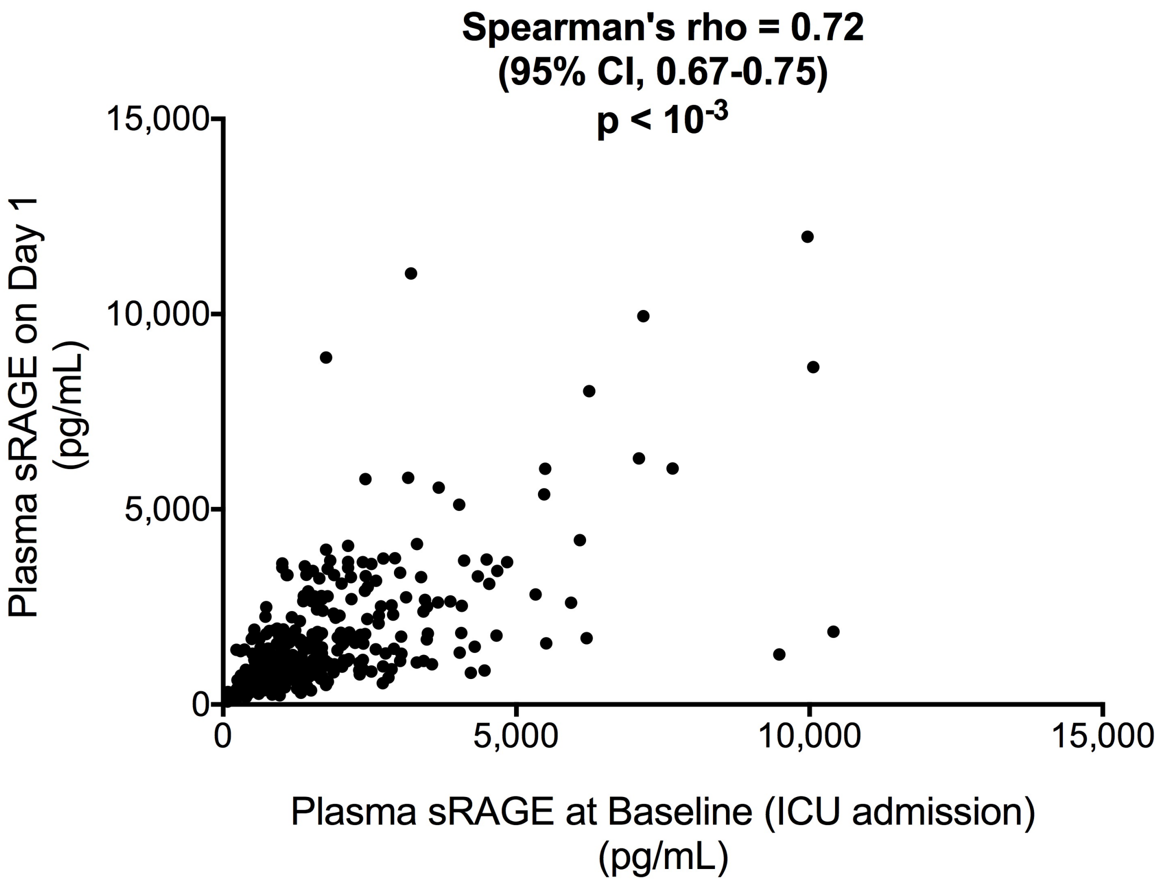

Supplementary Fig. S2. The three-dimensional structure of RAGE protein, A) without or B) with RAGE gene variant rs2070600A (Gly82Ser). No obvious changes in tridimensional structure were observed in RAGE tridimensional structure, using JavaScript-Based Molecular Viewer JSmol (http://jmol.sourceforge.net). White arrows indicate the position of the Glycine (Gly) or Serine (Ser) amino acid.

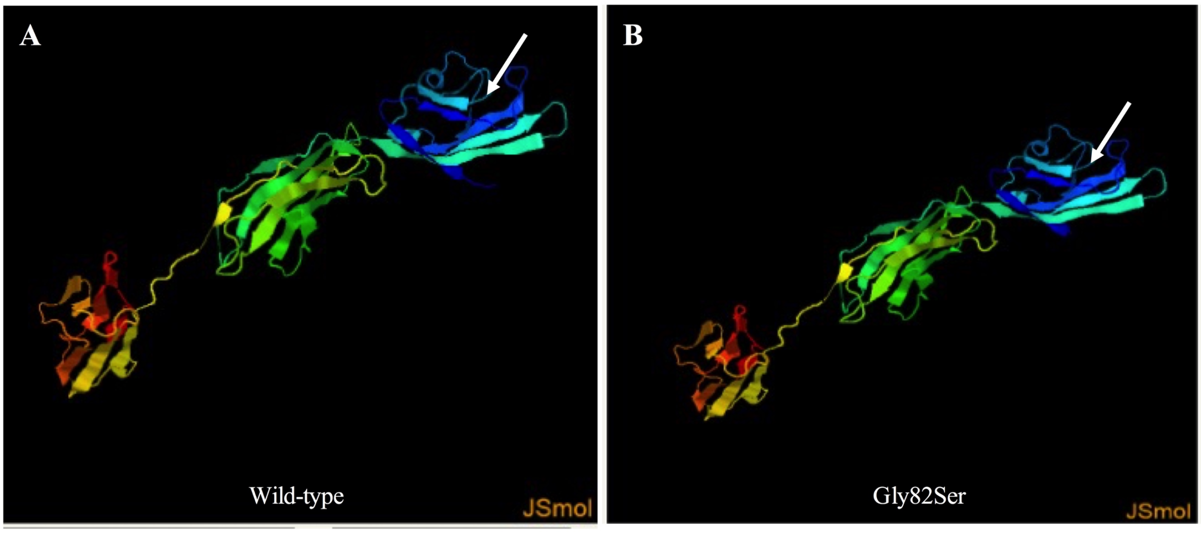

SUPPLEMENTARY TABLES

SNP	All patients (n = 464)		No ARDS (n = 405)		Develop ARDS (n = 59)	
	MAF	Estimated disequilibrium coefficient	MAF	Estimated disequilibrium coefficient	MAF	Estimated disequilibrium coefficient
rs1800625	0.14	0.01	0.13	0.01	0.14	0.02
rs1800624	0.27	0.02	0.29	0.02	0.28	0.04
rs3134940	0.14	0.003	0.14	0.02	0.15	0.04
rs2070600	0.06	0.03	0.06	-0.0003	0.08	0.03

Table S1. Single nucleotide polymorphisms (SNPs) within the *AGER* gene in all patients (n = 464), those who did not develop acute respiratory distress syndrome (ARDS) (n = 405), and those who developed ARDS (n = 59) by day seven. *MAF: minor allele frequency. The estimated disequilibrium coefficient gives the proportion of homozygotes in excess of what would be expected under Hardy-Weinberg equilibrium.*

Models	OR	95% CI	P value
**Baseline sRAGE**	2.39	[1.65–3.45]	$<10^{-3}$
**SNP rs2070600A (Ser/Ser genotype)**	124.59	[14.89–1,043.34]	$<10^{-3}$
SAPS II	1.01	[0.99–1.03]	0.1
Sepsis	1.68	[0.78–3.64]	0.2
Shock	0.93	[0.44–1.99]	0.9
Pneumonia	1.53	[0.62–3.79]	0.4

Table S2. The associations between plasma sRAGE, the *AGER* gene variant rs2070600A (the Ser/Ser genotype), and the prediction of ARDS by day seven in multivariate analyses. Analyses were adjusted for baseline severity (as assessed by SAPS II) and the presence of sepsis, shock, or pneumonia at baseline. Plasma sRAGE levels (in pg/mL) are natural log-transformed in the logistic regression model to meet the assumption of linearity with log-odds of outcome; the ORs presented here are for each log increase in the level of plasma sRAGE.

Models	Hazard ratio	95% CI	P value
**Baseline sRAGE ≥ 1,033 pg/mL**	4.59	[1.47–14.37]	0.01
**SNP rs2070600A (Ser/Ser genotype)**	15.09	[6.33–35.99]	$<10^{-3}$
SAPS II	1.01	[0.99–1.02]	0.2
Sepsis	1.41	[0.99–1.99]	0.05
Shock	1.03	[0.61–1.74]	0.9
Pneumonia	1.56	[0.82–2.98]	0.2

Table S3. The associations between plasma sRAGE, the *AGER* gene variant rs2070600A (the Ser/Ser genotype), and the prediction of ARDS by day seven in multivariate analyses. Analyses were adjusted for baseline severity (as assessed by SAPS II) and the presence of sepsis, shock, or pneumonia at baseline. The cutoff value of 1,033 pg/mL was chosen because it was the median value of baseline plasma sRAGE (in pg/mL) in our cohort.

Models	OR	95% CI	P value
**Baseline sRAGE**	2.25	[1.16–4.37]	0.02
**SNP rs2070600A (Ser/Ser genotype)**	15.52	[1.48–162.40]	0.02
Tidal volume	0.79	[0.48–1.30]	0.4
PEEP	0.96	[0.71–1.29]	0.8
Pplat	1.04	[0.91–1.19]	0.6
PaO_2/FiO_2	0.99	[0.98–0.99]	0.03
SAPS II	0.98	[0.94–1.02]	0.3
Sepsis	1.44	[0.25–8.40]	0.7
Shock	1.57	[0.42–5.91]	0.5
Pneumonia	1.14	[0.12–10.45]	0.9

Table S4. The associations between plasma sRAGE, the *AGER* gene variant rs2070600A (the Ser/Ser genotype), and the prediction of ARDS by day seven after multivariate analyses in patients under invasive mechanical ventilation (n = 222). Analyses were adjusted for tidal volume (in mL/kg predicted body weight), positive end-expiratory pressure (PEEP), inspiratory plateau pressure (Pplat), arterial oxygenation (the partial pressure of arterial oxygen [PaO_2] to the fraction of inspired oxygen [FiO_2] ratio), baseline severity (as assessed by SAPS II), and the presence of sepsis, shock, or pneumonia at baseline. Plasma sRAGE levels (in pg/mL) are natural log-transformed in the logistic regression model to meet the assumption of linearity with log-odds of outcome; the ORs presented here are for each log increase in the level of plasma sRAGE.

Direct insult to the lung	Indirect insult to the lung
Pneumonia<br>Aspiration of gastric contents<br>Inhalational injury<br>Pulmonary contusion<br>Pulmonary vasculitis<br>Drowning	Non-pulmonary sepsis<br>Major trauma<br>High-risk surgery<br>Pancreatitis<br>Severe burns<br>Non-cardiogenic shock<br>Drug overdose<br>Multiple transfusions or transfusion-associated acute lung injury (TRALI)

Table S5. The common risk factors for ARDS used to select at-risk patients in the PrediRAGE study.

SNP	Region	SNP sequence	Primers for PCR (5’□3’)	Tm	Sequencing Primer
**rs1800625**<br>*(_429 T/C)*	promoter	AAAAAAATG<br>ATTTTCTTTC<br>ACGAAG**[C/T]**<br>TCCAAACAGG<br>TTTCTCTCCT<br>GTTCC	*Forward*:<br>TCCTCACTTGTAAACT<br>TGTGTAG (23b)<br><br>*Reverse*:<br>TCTAGGGTCTCATTCC<br>CTCAG (21b)	64°C	*Forward*:<br>TGTAAACTTGTGT<br>AGTTTCAC (21b)<br><br>*Reverse*:<br>TCAGTCCATCAGG<br>GCTGCCTG (21b)
**rs1800624**<br>*(_374 T/A)*	promoter	GCCTTCATGA<br>TGCAGGCCCA<br>A[**T/A**]TGCAC<br>CCTTGCAGAC<br>AACA			
**rs3134940**<br>*(_2184 A/G)*	intron 8	TTCTTCCCTCT<br>GAGCTAAAA<br>AAAGG[**A/G**]A<br>CAGACGGCTG<br>GGCGCGGTGG<br>CTCA	*Forward*:<br>CAGCACAGGCTCTAAT<br>TTCCTG (22b)<br><br>*Reverse*:<br>GGCATGTGCCACCATG<br>CCTG (20b)	66°C	*Forward*:<br>TCTGGCCTTATCCC<br>TAACAG (20b)<br><br>*Reverse*:<br>TAGTAGAAATGGG<br>GTTTCTC (20b)
**rs2070600**<br>*(Gly82Ser)*	exon 3	CAGTGTGGCT<br>CGTGTCCTTC<br>CCAAC[**A/G**]G<br>CTCCCTCTTC<br>CTTCCGGCTG<br>TCGG	*Forward*:<br>AGACCAGCAATGATTT<br>GGATCC (22b)<br><br>*Reverse*:<br>ATGGGCCAAGGCTGG<br>GGTTG (20b)	64°C<br><br>66°C	*Forward*:<br>AGGCCTTGCACTG<br>TTTAGGC (20b)<br><br>*Reverse*:<br>AGACACGGACTCG<br>GTAGTTG (20b)

Table S6. The primers of the four variants of the *AGER* gene and their PCR and sequencing conditions. *SNP: single nucleotide polymorphism. PCR: polymerase chain reaction. Tm: temperature of melting. A: adenine. C: cytosine. G: guanine. T: thymine.*

STROBE Statement—Checklist of items that should be included in reports of ***cohort studies***

	Item No	Recommendation	Page(s)
**Title and abstract**	1	(*a*) Indicate the study's design with a commonly used term in the title or the abstract	Title page
		(*b*) Provide in the abstract an informative and balanced summary of what was done and what was found	Page 2
**Introduction**			
Background/rationale	2	Explain the scientific background and rationale for the investigation being reported	Page 3
Objectives	3	State specific objectives, including any prespecified hypotheses	Page 3
**Methods**			
Study design	4	Present key elements of study design early in the paper	Pages 12-13
Setting	5	Describe the setting, locations, and relevant dates, including periods of recruitment, exposure, follow-up, and data collection	Pages 12-13
Participants	6	(*a*) Give the eligibility criteria, and the sources and methods of selection of participants. Describe methods of follow-up	Pages 12-13-14
		(*b*) For matched studies, give matching criteria and number of exposed and unexposed	NA
Variables	7	Clearly define all outcomes, exposures, predictors, potential confounders, and effect modifiers. Give diagnostic criteria, if applicable	Pages 12-13-14
Data sources/ measurement	8*	For each variable of interest, give sources of data and details of methods of assessment (measurement). Describe comparability of assessment methods if there is more than one group	Pages 12-13
Bias	9	Describe any efforts to address potential sources of bias	Pages 14-15-16

Study size	10	Explain how the study size was arrived at	Page 14
Quantitative variables	11	Explain how quantitative variables were handled in the analyses. If applicable, describe which groupings were chosen and why	Page 14
Statistical methods	12	(*a*) Describe all statistical methods, including those used to control for confounding	Pages 14-15-16
		(*b*) Describe any methods used to examine subgroups and interactions	Pages 14-15-16
		(*c*) Explain how missing data were addressed	Page 14-15-16
		(*d*) If applicable, explain how loss to follow-up was addressed	Pages 14-15-16
		(*e*) Describe any sensitivity analyses	Page 15-16
**Results**			
Participants	13*	(a) Report numbers of individuals at each stage of study—eg numbers potentially eligible, examined for eligibility, confirmed eligible, included in the study, completing follow-up, and analysed	Page 4 and figure 1
		(b) Give reasons for non-participation at each stage	Figure 1
		(c) Consider use of a flow diagram	Done (Figure 1)
Descriptive data	14*	(a) Give characteristics of study participants (eg demographic, clinical, social) and information on exposures and potential confounders	Page 4 and table 1
		(b) Indicate number of participants with missing data for each variable of interest	Table 1
		(c) Summarise follow-up time (eg, average and total amount)	Page 4 and table 1
Outcome data	15*	Report numbers of outcome events or summary measures over time	Page 4 and table 1
Main results	16	(*a*) Give unadjusted estimates and, if applicable, confounder-adjusted estimates and their precision (eg, 95% confidence interval). Make clear which confounders were adjusted for and why they were included	Pages 4-5-6<br>Table 2<br>Tables S1-S2 (supplementary information)

		(*b*) Report category boundaries when continuous variables were categorized	Pages 4-5-6<br>Table 1<br>Figure 2<br>Tables S2-S3-S4 (supplementary information)
		(*c*) If relevant, consider translating estimates of relative risk into absolute risk for a meaningful time period	Page 6 and figure 4
Other analyses	17	Report other analyses done—eg analyses of subgroups and interactions, and sensitivity analyses	Pages 6-7
**Discussion**			
Key results	18	Summarise key results with reference to study objectives	Page 7
Limitations	19	Discuss limitations of the study, taking into account sources of potential bias or imprecision. Discuss both direction and magnitude of any potential bias	Pages 10-11
Interpretation	20	Give a cautious overall interpretation of results considering objectives, limitations, multiplicity of analyses, results from similar studies, and other relevant evidence	Pages 7-8-9
Generalisability	21	Discuss the generalisability (external validity) of the study results	Pages 8-9-10-11
**Other information**			
Funding	22	Give the source of funding and the role of the funders for the present study and, if applicable, for the original study on which the present article is based	Additional information (Page 25)

*Give information separately for exposed and unexposed groups.

Note: An Explanation and Elaboration article discusses each checklist item and gives methodological background and published examples of transparent reporting. The STROBE checklist is best used in conjunction with this article (freely available on the Web sites of PLoS Medicine at http://www.plosmedicine.org/, Annals of Internal Medicine at http://www.annals.org/, and Epidemiology at http://www.epidem.com/). Information on the STROBE Initiative is available at http://www.strobe-statement.org.
